# Improving Employee Voice About Transgressive or Disruptive Behavior: A Case Study

**DOI:** 10.1097/ACM.0000000000002447

**Published:** 2018-09-11

**Authors:** Mary Dixon-Woods, Anne Campbell, Graham Martin, Janet Willars, Carolyn Tarrant, Emma-Louise Aveling, Kathleen Sutcliffe, Janice Clements, Michelle Carlstrom, Peter Pronovost

**Affiliations:** 1 **M. Dixon-Woods** is Health Foundation Professor of Healthcare Improvement Studies and director, THIS Institute (The Healthcare Improvement Studies Institute), University of Cambridge, Cambridge, United Kingdom.; 2 **A. Campbell** is research associate, Division of Infectious Diseases, Imperial College, London, United Kingdom.; 3 **G. Martin** is director of research, THIS Institute (The Healthcare Improvement Studies Institute), University of Cambridge, Cambridge, United Kingdom.; 4 **J. Willars** is visiting research fellow, Department of Health Sciences, University of Leicester, Leicester, United Kingdom.; 5 **C. Tarrant** is associate professor, Department of Health Sciences, University of Leicester, Leicester, United Kingdom.; 6 **E.L. Aveling** is research scientist, Harvard T.H. Chan School of Public Health, Boston, Massachusetts.; 7 **K. Sutcliffe** is Bloomberg Distinguished Professor of Business and Medicine, Johns Hopkins University, Baltimore, Maryland.; 8 **J. Clements** is Mary Wallace Stanton Professor of Faculty Affairs and vice dean of faculty, Johns Hopkins University School of Medicine, Baltimore, Maryland.; 9 **M. Carlstrom** is founder, Safe at Hopkins, Johns Hopkins University, and principal consultant and executive coach, Build a Better Culture, Baltimore, Maryland.; 10 **P. Pronovost** is adjunct professor, Johns Hopkins University School of Medicine, Baltimore, Maryland.

## Abstract

**Purpose:**

Employee voice plays an important role in organizational intelligence about patient safety hazards and other influences on quality of patient care. The authors report a case study of an academic medical center that aimed to understand barriers to voice and make improvements in identifying and responding to transgressive or disruptive behaviors.

**Method:**

The case study focused on an improvement effort at Johns Hopkins Medicine that sought to improve employee voice using a two-phase approach of diagnosis and intervention. Confidential interviews with 67 individuals (20 senior leaders, 47 frontline personnel) were conducted during 2014 to diagnose causes of employee reluctance to give voice about behavioral concerns. A structured intervention program to encourage voice was implemented, 2014–2016, in response to the findings.

**Results:**

The diagnostic interviews identified gaps between espoused policies of encouraging employee voice and what happened in practice. A culture of fear pervaded the organization that, together with widespread perceptions of futility, inhibited personnel from speaking up about concerns. The intervention phase involved four actions: sharing the interview findings; coordinating and formalizing mechanisms for identifying and dealing with disruptive behavior; training leaders in encouraging voice; and building capacity for difficult conversations.

**Conclusions:**

The problems of giving voice are widely known across the organizational literature but are difficult to address. This case study offers an approach that includes diagnostic and intervention phases that may be helpful in remaking norms, facilitating employee voice, and improving organizational response. It highlights specific actions that are available for other organizations to adapt and test.

Health care is highly vulnerable to failures of organizational intelligence about hazards that may cause harm to patients,^1^ yet, as in other sectors,^2–4^ valuable sources of information about risks and about what may be going wrong are often undervalued.^5^ Employees are among the most important sources of “soft intelligence” about issues of poor care, faulty systems, or inappropriate conduct that can harm patients.^6^ Yet problems of silence (employees do not speak) and deafness (organizations do not hear or act) remain pervasive,^7^ especially (though not only) in the face of transgressive or disruptive behaviors by colleagues.^8^

A literature on employee voice characterizes the voicing of concerns as the discretionary disclosure of information intended to improve organizational functioning to someone with the perceived authority to act,^9^ but also makes clear that enabling and acting on voice are not straightforward.^10^ At the sharp end, hierarchies and professional boundaries^11^ and concerns about the potential to damage relationships^12^ may inhibit voice. A tendency toward “comfort-seeking” rather than “problem-sensing” behaviors^13^ at the leadership level—the so-called blunt end of care—may further stifle voice.

These challenges may be especially prominent when the issue of concern is transgressive or disruptive behaviors by powerful individuals, including misconduct, incivility, unreasonableness, bullying, harassment, and disrespect.^14^ Such behaviors are known to have profoundly negative effects on performance and culture.^15,16^ Allowing such behaviors to go unsanctioned may have deeply negative effects,^17^ interfering with employees’ psychological safety,^18^ undermining joint endeavors, increasing employee turnover, frustrating efforts to learn and improve, increasing costs,^19^ and ultimately increasing risks to patients. Health care is now recognizing that a major responsibility of system leaders is that of fostering climates and cultures of respect—in part encouraged by the realization that high-reliability organizations in other sectors do likewise.^20^ The first step, of course, is gaining knowledge of the problems. The second is doing something about them. Neither is easy, but both are essential.^21^

In this article, we describe how Johns Hopkins Medicine (JHM), a large academic medicine entity, sought to improve its ability to detect and manage patient safety concerns, including transgressive/disruptive behaviors, and to mount a program of improvement.

## Method

We report a case study^22^ of an improvement effort that involved two phases: a diagnostic phase, involving interviews with JHM personnel conducted by an independent team of evaluators, and an intervention phase that responded to the diagnostic findings.

### Diagnosis phase: Interview study

Following discovery of serious misconduct by a physician, JHM commissioned a team of evaluators (authors M.D.W., G.M., J.W., C.T., A.C., and E.L.A.) from a UK university to explore influences on employee voice through a qualitative interview study. In early 2014, heads of five purposively selected departments across the JHM system that represented the majority of faculty and staff (medicine, surgery, neurology, pediatrics, and anesthesiology and critical care medicine) were asked to distribute an e-mail asking personnel at all levels to take part in confidential, anonymized interviews. A separate e-mail from the senior vice president for quality invited senior leaders (e.g., departmental chairs, executives, board members) to participate. A secure link, which could not be monitored by anyone at JHM, was provided for direct response to the evaluation team. Every individual who responded and agreed to be interviewed participated in the study: No sampling within the respondents was undertaken.

Interviews were conducted during the spring of 2014 using a semistructured prompt guide that included questions about people’s general views and experiences of raising patient safety concerns and hypothetical scenarios that included transgressive or disruptive behaviors. Senior leaders were interviewed either in person or by telephone (by author G.M.); others were conducted, all by telephone (by J.W.). All interviews were audio-recorded and transcribed verbatim except that all identifying details were removed to ensure anonymity.

This study was submitted to the Johns Hopkins University Institutional Review Board and was deemed Quality Improvement. Consent was taken orally to avoid creating a written record of individuals’ participation. No transcripts were shared with JHM.

Data analysis was based on the constant comparative method.^23^ Ten interviews were open-coded line-by-line (by A.C.). The open codes, together with sensitizing constructs derived from the literature, were used (by A.C., M.D.W., and G.M.) to develop an initial coding frame which was applied to subsequent interview transcripts, and was iteratively refined until all the transcripts were analyzed. NVivo qualitative software, version 10 (QSR International Pty, Ltd., Australia), was used to manage the coding and analysis process.

In presenting our findings, we attribute quotations to either frontline (FL)—people directly involved in care delivery or in supporting care—or senior leader (SL) participants. Sometimes details of quotations have been disguised to ensure confidentiality.

### Phase 2: Intervention phase

An anonymized report of the interview study was shared with the JHM leadership team in July 2014. The actions that JHM leaders (including P.P., J.C., and M.C.) took in response over the following two years (up to summer 2016) were shared verbally and through notes and documentation with the evaluation team, who summarized the material and checked back understanding. We present the account of these actions descriptively below.

## Results

For the diagnostic phase, we organized our analysis around the themes of the untouchables; the gap between policy and practice; whether it was safe to speak; whether it was worth speaking up; and responding to and acting on the findings. For the intervention phase, the themes included publicly sharing the study findings; coordinating and formalizing mechanisms to identify, assess, and remedy disruptive behavior; training in leadership behaviors to encourage voice; building capacity to have difficult conversations; and impact.

### The diagnostic phase

Reponses to the invitation to interview were received from 118 personnel (91 frontline and 27 senior leaders). We interviewed everyone with whom it was possible to arrange an interview (47 frontline and 20 senior leaders: 67 in total). Because of the nature of the distribution mechanism, it was not possible to determine a precise response rate. Many participants reported how much they appreciated the opportunity to describe their experiences and perceptions, suggesting that the interviews themselves may have been a valuable intervention.

#### The untouchables.

Many of the challenges of giving voice at JHM were personified in the widely discussed problem of the “untouchables”: individuals, usually senior physicians, who appeared to engage in transgressive or disruptive conduct with impunity.

A lot of times they’re just real jerks, so they’re just mean people—they just yell at people and they think that they can do whatever they want basically. (SL)

The untouchables’ ability to get away with egregious behavior was reported to derive from their positions of power within the organization or their capacity for revenue generation, or both.

It turns out after discussion amongst ourselves that he’s sort of one of these untouchables. It’s very hard to discipline him, we can’t ever get him to comply, he does whatever he wants and sometimes people are afraid to confront, particularly the surgeons and physicians that are seen as being big revenue generators. (SL)

The effects of these kinds of behaviors were toxic. Direct breaches of safety rules clearly represented a primary risk of harm. But the secondary effects were, in many ways, at least as important: The untouchables contributed to tense, conflict-laden, oppressive working environments that constrained their colleagues’ ability to take joy or pride in their work, with all the attendant risks of such a context—including poor teamwork and difficulties in staff retention.

The scrub nurse came out of the operating room very flustered. She said, “There’s only so much I can do, I told him to put on his isolation gown and he just refused to do it, he’s not doing it and I can’t—I can only push it so far.” (SL)

Unsanctioned rule breaches risked normalization of deviance,^24^ especially when more junior colleagues looked to the untouchables as role models. The failure to deal with the problems contributed to an erosion of trust in senior management and was a major feature of the culture of fear that pervaded the organization. An illustrative remark was:

Until very recently, there has been very weak leadership of this area. It wouldn’t address problems anyhow. I think [the leadership of] the department is best described as a bunch of cowboys. (SL)

#### The gap between policy and practice.

Though senior leaders emphasized the need for concerns about patient safety and quality of care to be raised, and described multiple mechanisms to support this, the reality was that the organization lacked coordinated processes to identify, assess, and remedy disruptive physician behaviors. Not everyone in the organization knew of the procedures to report concerns, and the systems were tedious, complex, unreliable, and lacking in clarity for accountability. Information was reported through many different channels (e.g., error reporting, human resources) that varied in their definitions of disruptive behavior, in their procedures, and in their impact.

They make it very difficult to report your managers. I mean I would still do it, but all the protocols and red tape […] make it very difficult for the person who is going through the bad experience [to] get some sort of satisfaction. (FL)

#### Is it safe to speak?

Although participants reported some positive experiences in voicing concerns, more common was the finding that organizational silence was linked to an underlying culture of fear. Participants reported that fear permeated the organization and hindered personnel at all levels—from the most junior to the most senior—from reporting concerns that might challenge the interests of others or disrupt the status quo. About a colleague, one participant observed:

He wasn’t going to raise [the concern] because it would take the project off the timeline … because the pressure of the leadership and colleagues and everybody would be, “What is the matter with you? You’re not a team player, don’t you get it?” That’s it. It’s a generalized pressure to stay in your box—it’s not your business—and conform. And it’s really, really, really deep and nobody is going to step out of it and nobody does. (SL)

The reasons for this fearfulness were varied. The dynamics of power and blaming behavior, intent on the defense of positions and territories, meant that people were afraid that challenging powerful individuals and their allies would limit their careers, that raising concerns would escalate into conflict and recrimination, that they would be labeled as a snitch, a whiner, a poor performer or an alarmist, or that they could be subject to retaliation. For instance:

You only have to be on the other end of a lashing before you decide you’re not going to do that again. And so going one-on-one and trying to get it resolved, that’s not part of what we do in the culture. Having been the recipient of it a number of times, it’s very risky. (SL)

Other fears centered on the risk that raising concerns could result in exposure to litigation, prolonged and draining conflict with no assurance of success, or accusations of discriminatory behavior.

I should really speak up and say something here, but number one, I know from history that it’s not going to be well received, and I know that it’s going to end up putting me in a worse position and that’s not going to accomplish anything. (FL)

Those who thought themselves to be at some kind of disadvantage—perhaps by being new or in a hierarchically inferior position—were seen by participants as especially nervous about speaking, as this remark illustrates:

Residents felt it was academic suicide to ever question or raise an issue with an attending. (SL)

#### Is it worth speaking up?

People’s beliefs about the likely effects of giving voice to their concerns strongly influenced their inclination to speak. Understandably, having a positive experience appeared to reinforce willingness to voice concerns, and many encouraging examples were reported. But participants also reported hostile responses to attempts to raise concerns. For example:

[The doctor] didn’t see the patient. He became verbal, very irate. So I just walked away. It wasn’t doing any good and I had approached him quietly but it did no good. (FL)

Perhaps more common than hostile responses were nonresponses. Senior leaders and those at the sharp end perceived that their concerns were not always taken seriously or that nothing happened. Reluctance by senior management to grasp long-standing thorny problems and a lack of organizational commitment and capacity for problem solving were described, resulting in difficult situations persisting over long periods of time without being remedied. Two observations are illustrative:

They’ve been frustrated by things for so long that [they think] why bother? (SL)

Again, it’s a leadership issue; they [don’t] pay attention or just pooh-pooh it instead of seriously addressing it. (FL)

People reported that they sometimes simply stopped raising concerns, because to do so was irrational: It invited risk without any prospect of benefit. In consequence, many at the sharp end expressed frustration that although the organization invited staff and faculty to speak about concerns, it appeared to lack an authentic capacity for listening or a full commitment to address concerns.

[There are] pockets of historically weak leadership where we learn that there have been ongoing issues for years that people [managers] have been either unwilling or uncomfortable addressing. So after a while you just stop talking about it. (SL)

### The intervention phase: Responding to and acting on the findings

The first step of the intervention phase involved the evaluation team reporting the findings of the interview study in report form (summer 2014) and through presentations to JHM leaders (fall 2014–spring 2015). In response, leaders instituted a structured program of improvement involving four key actions: publicly sharing the findings; coordinating and formalizing mechanisms for identifying and dealing with disruptive behavior; training leaders in how to encourage voice; and building capacity to have difficult conversations. Much of this organizational response was informed by the literature suggesting that voice behaviors (speaking out to peers and up to supervisors) increase when employees are provided greater opportunities to give voice and when they believe that their input will make a difference, known as “voice instrumentality.”^25^

#### 1. Publicly share the study findings.

The findings of the study were shared publicly with stakeholder groups across the JHM system. In spring 2015, the evaluation team personally presented the study results at the JHM Patient Safety and Quality Board meeting, targeting senior leaders, as well as at town hall meetings to which all personnel (especially those at the sharp end) were invited, with large attendance. In addition, senior leaders from JHM presented the results in multiple forums, including to departmental directors, departments, and nursing groups, throughout 2015.

This widespread sharing of the discomfiting findings had a marked effect, with many remarking that it signaled a new openness and a willingness on the part of senior leadership to listen to concerns. One immediate impact was that several staff members came forward with new confidence to report colleagues with chronic and severe disruptive behavioral problems. A typical comment was, “You say you want to hear about untouchables; I want to share my concerns to see if you are serious.”

#### 2. Coordinate and formalize mechanisms to identify, assess, and remedy disruptive behavior.

A major response to the findings of the interview study was to coordinate and formalize the multiple initiatives and mechanisms for raising concerns. Many initiatives had been under way since 2010–2012 but were operating in silos. Improved coordination and communication was seen as critical to rationalizing and professionalizing the multiple channels and ensuring appropriate action.

A critical feature of the newly integrated approach was a program known as Safe at Hopkins, which had been in development since 2011, had had a “soft launch” in 2013, began to operate broadly in 2014–2015, and upgraded its website in 2015. Developed out of workplace violence prevention initiatives by the leader of the JHM risk assessment team and other behaviorally trained colleagues, it was purposefully designed to work outside the formal faculty and hierarchical structures. It sought to promote a culture of safety through prevention and early intervention in unprofessional, transgressive, and disruptive behaviors, and was specifically positioned to provide psychological safety. It collects concerns about disruptive, bullying, or violent behavior and any impact on the workplace, and where appropriate, may conduct an in-depth review of the concerns. It uses a structured interview approach to classify behaviors in order to develop themes and make recommendations. Team members are explicit in initial conversations that they are exploring potentially concerning behavior without specifically identifying any individual; they speak broadly with students, residents, staff, faculty, and administrators and aim to offer a safe place to discuss concerns and options for addressing them.

**Figure 1:**
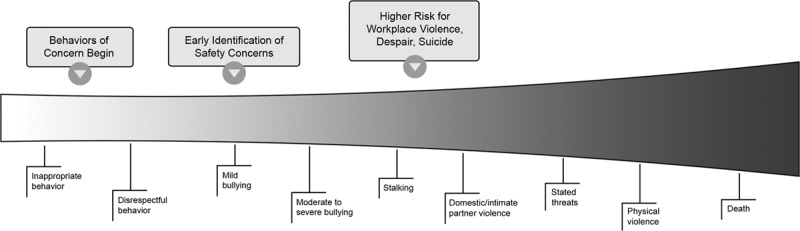
Johns Hopkins continuum of disruptive behaviors at work, from a study of improving employee voice about disruptive and transgressive behavior, Johns Hopkins Medicine, 2014–2015.

Where needed, Safe at Hopkins works in partnership with a high-level leader within JHM to remediate individuals’ behavior, initially through communication that emphasizes the impact of disruptive behavior on a culture of safety and the working climate. Individuals may also be referred for coaching or other skill building. But if behavior does not improve, the leader may cite the Safe at Hopkins review and remove the person from her/his position of power. Though the HR processes are different for different categories of employees, the system applies to all faculty and staff. To promote a collective understanding and common language, disruptive behaviors were described explicitly, supported by the Johns Hopkins continuum of disruptive behaviors at work (Figure 1), which identified unwanted behaviors, providing explanations and examples. A flowchart of various types of behavior that must be referred to specific offices (e.g., for legal reasons) was also developed, and a checklist was provided to departmental leaders.

A further key element of the newly coordinated approach to raising concerns was the creation of the Physician Executive Oversight Committee (PEOC), which includes leaders from the Johns Hopkins University School of Medicine (faculty employer) and the Johns Hopkins Health System (employer of staff and nonfaculty physicians). Its work is protected from discovery in legal proceedings. Meeting quarterly, it communicates and coordinates information, triangulating data from multiple sources, including the incident reporting system, employee engagement surveys, informal reports, Safe at Hopkins, and others. The PEOC does not itself have investigatory or disciplinary remit. However, a signal deemed sufficiently severe or chronic may result in the vice dean’s office or other leader initiating a review and potentially issuing sanctions.

#### 3. Training in leadership behaviors to encourage voice.

Organizational research suggests that leaders’ behaviors and the structures they create are central to shaping cultures that facilitate speaking up.^25^ In addition to sharing the results of the diagnostic phase widely with leaders across JHM, leaders were, shortly after the feedback from the diagnostic interviews, provided with training and tools regarding voice opportunities, including how their staff could report concerns. The process for identifying and investigating disruptive behaviors was discussed at board and leadership meetings. Leaders were given advice about coaching to enable them to facilitate voice instrumentality—for example, by creating psychological safety to report and ensuring zero tolerance for retaliation.

#### 4. Build capacity to have difficult conversations.

Following the interview study, senior leaders met with departmental directors to better understand directors’ barriers to addressing disruptive physicians more effectively. Department directors and medical staff leaders welcomed the findings of the interview study in exposing the challenges they faced, and further welcomed the Safe at Hopkins program as it provided them “cover and support.” But many described their discomfort in raising concerns with particular individuals. An external expert was engaged to develop a course with a 30-minute e-learning module followed by a two-hour interactive simulation workshop in summer 2015 in which leaders practiced having difficult conversations.

### Impact of the intervention phase

Though not yet evaluated formally, the interventions seem to be having an impact. Over a two-year period from the time the problem of “untouchables” came to prominence after the interview study (2014), the Safe at Hopkins program consulted on 382 reports of disruptive behavior and conducted 55 in-depth reviews involving over 400 interviews, including 20 reviews of individuals in superior positions to those affected by the behavior. The vast majority of reported individuals were referred for additional support. Nine were eventually removed from their positions of power.

Decisions are confidential, but the changes in a physician’s role in the institution are not. The Safe at Hopkins program has received positive responses from staff—for example, “I never thought I would see the day when Hopkins leaders addressed this person’s behavior; thank you; perhaps we have a culture change occurring.” One manager, after attending an open town hall meeting about the interview study, submitted a concern about a “chronic untouchable” who had significant power in the organization. An investigation was then initiated and the individual moved. The manager responded: “I have been at Hopkins 20 years, and never thought I would see the day when this type of behavior is not tolerated. You have done more to restore my faith in leadership with this action than all of you have done over the last 20 years, thank you.”

## Discussion

Though the problem of disruptive behaviors is widely described in the literature, it is rarely easy to address.^26,27^ Diagnostic interviews showed that two prominent features of the culture at JHM were implicated in reluctance to speak: first, a hierarchical culture pervaded by a generalized fearfulness at almost every level, where territories and autonomies were often fiercely defended by powerful individuals and their allies; and second, uncertainties about whether positive action would result from the exercise of voice. The problem known colloquially as “untouchables”—individuals who engaged with impunity in transgressive or disruptive behavior—corrupted the conditions of a healthy working environment, resulting in personnel feeling fearful and lacking in psychological safety.^28^ An especially unfortunate consequence was that failure to tackle these problems eroded trust in senior management.

Little of what was found at JHM in the interviews is unusual. The problems of exercising voice challenge most major industries worldwide.^29–31^ Through the goals they set, where they focus their attention, their communications and information sharing, the structures and programs they create, and other decisions and actions, leaders shape cultures by signaling what is and is not important, and how organizational members should act and interpret events,^32^ while employees make sense of the overall pattern of signals sent by organizational leaders.^33^ The interviews showed that employees made sense of discrepancies between espoused and enacted priorities for raising concerns, and in so doing socially verified what they thought really mattered. This is why the second, interventional phase was vital in demonstrating JHM’s willingness to confront the issues and take action to reduce the risks to patients associated with silence, deafness, and inaction.

Some elements of what we describe here, including Safe at Hopkins, share characteristics with other professionalism initiatives,^34^ suggesting emerging consensus on the need for structured processes for identifying and acting on concerns for individuals. The approach as a whole, from diagnostics through to intervention, is more than any single element, however. The commissioning of the interview study and the wide public sharing of the findings functioned as an intervention in its own right, demonstrating to skeptical colleagues a new appetite for learning in the organization. It signaled that the gap between what was said and what was being done was narrowing. The subsequent action plan, the coordination of mechanisms for raising and responding to concerns, and the resulting removal of senior individuals from positions of power were evidence of important shifts in the attention given to hearing employee voice and the fresh commitment of the organization to tackle difficult problems. The approach thus supported the remaking of what Detert and Edmondson^10^ term “implicit voice theories” that result in people self-censoring at work.

We propose that the approach we describe here offers potentially generalizable principles (List 1) that could be evaluated in other contexts. The practical delivery of the program, of course, requires energy and resources. The Safe at Hopkins program is funded by Johns Hopkins Health System and Johns Hopkins University and currently employs one investigator who leads the program with administrative support. A (noncompensated) committee structure is also needed, though new IT systems are not. Perhaps most important, however, the approach requires courageous institutional leadership and a fearless commitment to organizational values and mission. The mandate for the initial interview study and subsequent actions came from the trustees of JHM and was implemented jointly by university and health system leadership.

This study has limitations. Owing to the confidential nature of the interviews, it was not possible to determine the representativeness of the interview participants; Johns Hopkins employs approximately 32,000 full- and part-time employees and hosts 12,000 graduate, professional, and medical students. No formal evaluation of the action plan has taken place, so it is not possible to draw firm conclusions about its impacts. Instead, these actions may be regarded as hypotheses for further testing, perhaps in other contexts. Future work should also include economic evaluation of the costs and benefits of this approach.

## Conclusions

Organizations that tolerate disrespectful behaviors will struggle to create the norms and values necessary to provide safe, patient-centered, and efficient care and a joyful work environment. If health systems are to deliver safe, high-quality care, and if health care employees are to feel respected and have joy in work, health system leaders (at all levels) need to demonstrate their commitment to hearing from their own personnel about safety concerns, and then respond authentically. The systematic efforts we have described here may provide useful principles and a model that can be evaluated in new contexts to help others address similar problems.

## List 1

A Testable Approach to Encouraging Voice in Relation to Disruptive Behaviors, From a Study of Improving Employee Voice About Disruptive and Transgressive Behavior, Johns Hopkins Medicine, 2014–2016

- Conduct diagnostics, for example, a confidential interview or survey study, to identify barriers to voice.
- Share the findings of the diagnostics at all levels to foster understanding and to signal openness and willingness to make change.
- Senior leaders declare and communicate a goal of encouraging voice, including (but not only) voice in relation to disruptive or transgressive behavior.
- Define the values and principles that should inform the conduct of all personnel, and specify behaviors that are deemed unacceptable (using a continuum if appropriate); ensure that these are understood at all levels of the organization.
- Ensure clear, well-coordinated mechanisms for reporting disruptive behavior, along with a facility for gathering “soft intelligence”; ensure that these are understood at all levels of the organization.
- Institute a well-founded investigation process, with defined and transparent processes for investigating concerns and triangulating data sources, that can be activated in cases of concern.
- Ensure that leaders understand the importance of addressing concerns, and provide training and development mechanisms to increase organizational capacity for action.
- Specify what will happen in response to disruptive or transgressive behavior, and ensure that these consequences are implemented consistently and effectively.
- Monitor the activities and outcomes of the program, act to ensure that they are achieving the intended objectives, and make visible the outcomes of the processes.

## 

*Acknowledgments:* The authors thank the staff, faculty, and board of the Johns Hopkins Health System.
